# Development of Plant-Based Leather from Naturally Dyed Banana Pseudo-Stem Using a Low-Energy Process

**DOI:** 10.3390/polym18101154

**Published:** 2026-05-08

**Authors:** Seranee Srisuk, Thanakorn Sodsai, Penwisa Pisitsak

**Affiliations:** 1 Department of Materials and Textile Technology, Faculty of Science and Technology, Thammasat University, Pathum Thani 12121, Thailand; seranee@tu.ac.th; 2 Baan Chang Sakul Baisri Community Enterprise, Ratchaburi 70120, Thailand; thanee.brand@gmail.com; 3 Center of Excellence on Petrochemical and Materials Technology, Chulalongkorn University, Bangkok 10330, Thailand

**Keywords:** banana pseudo-stem, natural dye, lac dye, indigo dye, plant-based leather, sustainable material

## Abstract

This study explores the natural dyeing of banana pseudo-stem (BSS) fibers as a sustainable material for plant-based leather applications through low-energy processes. Two dye types, lac dye and indigo blue, were applied, and the optimal dyeing parameters were systematically determined. Optimal lac dyeing was achieved using pre-mordanting with a binary mordant system comprising tannic acid and aluminum potassium sulfate (5 g L^−1^ each), a dye concentration of 10% owf, a pH of 4.23, and a dyeing duration of 6 h. For indigo dyeing, the optimal conditions involved 500 g L^−1^ wet indigo, 30 g L^−1^ sodium hydroxide, and 40 g L^−1^ thiourea dioxide, with a reduction step at 30 °C for 30 min. The dyed samples exhibited good to excellent perspiration fastness in terms of staining, while color change ratings remained low due to the intrinsic sensitivity of natural dyes. Light fastness was rated fair. The dyed BSS was subsequently laminated onto a polypropylene (PP) nonwoven substrate and coated with natural rubber (NR) latex, yielding bursting strengths of 51.8–54.8 psi. Moisture management testing confirmed inherent waterproof properties. Overall, this study presents a resource-efficient approach to transforming agricultural residues into sustainable, plant-based leather alternatives.

## 1. Introduction

At present, the world faces serious challenges related to climate change and environmental pollution, highlighting the importance of the circular economy concept. This approach emphasizes extending product lifespans through reuse and recycling before materials are discarded as waste. In parallel, the bioeconomy concept, which focuses on replacing petroleum-based resources with renewable bio-based raw materials for the production of a wide range of products, has gained increasing global attention [1]. In the long term, integrating circular economy and bioeconomy strategies can reduce environmental impacts and enhance sustainability. These transitions have significantly influenced various industrial sectors, including the textile and leather industries, which are under increasing pressure to adapt to changing consumer expectations [2]. Increasingly, consumers prioritize ethical considerations and sustainability as key factors in their purchasing decisions, thereby driving demand for environmentally responsible materials and production processes [3,4].

The leather industry is an economically important sector that generates substantial revenue, particularly from genuine leather such as cowhide, which is widely regarded as a valuable by-product of the meat industry [5], as well as from exotic leathers such as crocodile skin. Leather is commonly used in a wide range of products, including garments, footwear, handbags, belts, furniture, and automotive interiors [6,7,8]. However, conventional leather manufacturing processes, from rawhide preparation and tanning to dyeing, typically involve the extensive use of f hazardous chemicals, including formaldehyde, lead, cyanide, chromium compounds, and synthetic dyes [6,7,8]. Wastewater and solid waste generated from tanneries often contain these pollutants, resulting in air, water, and soil contamination and causing severe adverse impacts on ecosystems [5,8].

Artificial leathers are composite materials or laminates consisting of woven fabrics coated with polymers. For instance, polyester fabrics coated with polyvinyl chloride (PVC) or polyurethane (PU) are economical artificial leathers [6] that exhibit higher UV and moisture resistance than genuine leather. The manufacturing process of artificial leather also enables products with high surface uniformity [9]. However, conventional synthetic leather remains petroleum-derived, and PVC-based coatings often contain high proportions of plasticizers, which may raise potential health and environmental concerns [6].

As a result, so-called alternatives have emerged to replace petroleum-based artificial leather [6,10]. This approach utilizes natural fibers to produce vegan leather, which is designed to mimic the tactile feel, flexibility, and durability of natural leather. These materials have been fabricated by blending polymers, such as polylactic acid or natural rubber, with renewable fillers or fibers, including cork, pineapple leaf fiber (Piñatex^®^), apple pomace (AppleSkin^®^), cactus (Desserto^®^), and other plant residues. Fungi-based leathers developed from mycelium and mushrooms, such as Muskin^®^, as well as bacteria-based leathers produced from bacterial cellulose, have also been reported [11,12,13]. These plant-based leathers are generally considered eco-friendly and biodegradable [11]. Previous studies have reported the use of natural rubber coatings on pineapple leaf fibers. In particular, a recent study described the fabrication of green leather alternatives using natural rubber and pineapple leaf fiber (PALF) via a nonwoven mat formation and latex-coating process. The incorporation of 5% epoxidized natural rubber (ENR) enhanced tensile strength and interfacial adhesion, suggesting that such biocomposites have strong potential as sustainable substitutes for both animal and synthetic leathers [1].

Banana (*Musa* spp.) is a herbaceous plant belonging to the family Musaceae [14], and is native to South and Southeast Asia [15]. India is the world’s largest producer, accounting for approximately 31.5% of global banana production [16]. In the banana plantation industry, the banana pseudo-stem (BSS) is typically discarded after fruit harvesting, resulting in a substantial volume of agricultural waste. This BSS can be valorized as a source of natural fibers for the production of handmade paper, banana fiber textiles, garments, and vegan leather [14,17]. Previous studies have compared fiber extraction methods from BSS, such as the steam-release method and the enzymatic-release method using pectinase derived from *Staphylococcus sciuri*. Fibers obtained through enzymatic extraction exhibited superior quality and were considered highly suitable for producing organic and ecological textiles [18].

Natural dyes can be derived from various parts of plants, including roots, bark, leaves, flowers, and fruits, as well as from animals and minerals. They have been traditionally used for coloring food, leather, wood, and both natural fibers, such as wool, silk, cotton, and linen, and synthetic fibers, such as nylon [19,20,21]. Most natural dyes contain bioactive phytoconstituents, which enable the development of biofunctional textiles with antioxidant, antimicrobial, and UV-protective properties [22]. Thailand, located in a tropical region, is rich in plant biodiversity and therefore represents an important source of natural dye materials.

Indigo blue dye belongs to the class of indigoid colorants. It is an insoluble blue organic compound that occurs naturally in the leaves of *Indigofera tinctoria* and related species, which are commonly cultivated in tropical countries such as China, India, and Thailand [23]. Indigo blue dye exhibits a deep blue color and is insoluble in water [24]. Upon enzymatic hydrolysis, indican releases indoxyl, which subsequently undergoes oxidation to form insoluble indigo blue pigment [25]. For dyeing applications, indigo must be reduced in an alkaline medium to its water-soluble leuco-indigo form, which exhibits strong substantivity toward cellulosic fibers [23,24,26].

Lac dye is a natural red colorant obtained from the secretions of the lac insect, *Kerria lacca* (formerly *Tachardia lacca*). The dye is commercially known as C.I. Natural Red 25 (C.I. 75450). Female lac insects secrete a resinous substance during colonization of host tree branches. Lac dye is widely used for dyeing wool, silk, and cotton textiles. It is generally applied in combination with metallic mordants such as aluminum potassium sulfate (AlK(SO_4_)_2_), potassium dichromate (K_2_CrO_7_), ferrous sulfate (FeSO_4_), and stannous chloride (SnCl_2_) to improve dye–fiber affinity and colorfastness [27,28].

This study evaluated the dyeing characteristics of BSS sheets using bio-based dyes derived from indigo and lac under low-energy dyeing conditions. The dyeing parameters were systematically optimized for each dye system. The dyed BSS sheets were subsequently laminated with a polypropylene (PP) nonwoven substrate and coated with natural rubber (NR) latex to form a plant-based leather structure. The color properties, color fastness, moisture management behavior, and bursting resistance of the resulting materials were then evaluated to assess their suitability for plant-based leather applications.

## 2. Materials and Methods

### 2.1. Materials

Banana pseudo-stem (BSS) was sourced from Chet Samian Subdistrict, Ratchaburi Province, Thailand. Meanwhile, natural indigo dye and natural lac dye were obtained from Sakon Nakhon Province and Lampang Province, Thailand, respectively. Hydrogen peroxide (H_2_O_2_, analytical grade) was purchased from QReC, Auckland, New Zealand. Tannic acid (C_76_H_52_O_46_) was supplied by Loba Chemie Pvt. Ltd., Mumbai, India. Aluminum potassium sulfate dodecahydrate (AlK(SO_4_)_2_·12H_2_O) and sodium hydroxide (NaOH) were obtained from Kemaus Chemicals, Cherrybrook, New South Wales, Australia. Thiourea dioxide (CH_4_N_2_O_2_S, commercial grade) was purchased from Common Co., Bangkok, Thailand.

### 2.2. Preparation of BSS

The dried BSS sample was cut into 4 × 4 cm specimens. The color values of each specimen were determined, and the color difference values were calculated according to the CIEDE2000 color difference formula. A color difference value not exceeding 1 was considered acceptable (ΔE 2000 ≤ 1).

### 2.3. Chemical Composition Analysis of BSS

The chemical composition of the dried BSS sample was determined to quantify the main lignocellulosic components, including extractives, lignin, holocellulose, α-cellulose, and hemicellulose. The content of extractives was determined according to TAPPI T204 (alcohol–benzene solubility) and TAPPI T264 (alcohol and hot water solubility). Acid-insoluble lignin was measured following TAPPI T222. Holocellulose content was determined using the Browning method. The α-cellulose content was determined according to TAPPI T203, and hemicellulose content was calculated as the difference between holocellulose and α-cellulose. All measurements were performed in duplicate, and the average values are reported.

### 2.4. Morphological Analysis of BSS

The morphology of the BSS sample was examined using scanning electron microscopy (SEM) (JSM-5410LV, JEOL Ltd., Tokyo, Japan). The samples were mounted on aluminum stubs using double-sided conductive carbon tape and sputter-coated with a thin layer of palladium to enhance electrical conductivity. SEM observations were carried out at an accelerating voltage of 20 kV, and images were recorded at a magnification of ×200.

### 2.5. Lac Dyeing of BSS

Lac dyeing of BSS was performed using a cold pad–batch technique. BSS sheets were immersed in the lac dye solution for 10 min, followed by padding at a wet pick-up of 90–95% using a two-dip, two-nip procedure. The padded specimens were then sealed in zipper bags at a liquor ratio (L:R) of 1:30 and subsequently air-dried.

To examine the effect of dyeing parameters, cold pad–batch dyeing was conducted at batching times of 6, 12, 18, and 24 h. The lac dye concentration was varied at 5, 10, 20, and 30% owf. The pH of the dye bath was adjusted to 3, 4.23 (as-prepared dye solution), 5, 7, 9, and 11. Mordanting was carried out using tannic acid (T) and aluminum potassium sulfate (Al), either individually or in combination, at concentrations of 5 and 10 g L^−1^, denoted as T5, T10, Al5, Al10, T5Al5, T5Al10, T10Al5, and T10Al10.

### 2.6. Indigo Dyeing of BSS

A natural indigo vat was prepared by reducing indigo at 30 °C for 30 min. The dyed BSS samples were then immersed in the reduced indigo solution for 30 min, followed by squeezing and exposure to air to allow oxidation of leuco-indigo to its insoluble indigo form. This dyeing and oxidation cycle was repeated twice to enhance color development. The dyeing process was carried out at a liquor ratio (L:R) of 1:50.

To investigate the effects of dyeing parameters, the concentration of wet indigo was varied at 100, 200, 300, 400, 500, 700, and 900 g L^−1^. Thiourea dioxide, used as the reducing agent, was tested at concentrations of 10, 40, 60, 80, and 100 g L^−1^. The sodium hydroxide (NaOH) concentration was adjusted to 10, 20, and 30 g L^−1^ to control the alkalinity of the dye bath.

After dyeing, the samples were immersed in a 5% (*w*/*v*) hydrogen peroxide (H_2_O_2_) solution for 3 min to ensure complete oxidation. They were then rinsed thoroughly with water and air-dried under ambient conditions.

### 2.7. Preparation of Plant-Based Leather via Nonwoven Lamination and Natural Rubber Latex Coating

The dyed BSS sheets were used as the primary structural substrate. Each BSS sheet was laminated onto a polypropylene (PP) nonwoven fabric using a poly(vinyl acetate) (PVAc) latex adhesive. Subsequently, the exposed surface of the BSS layer was coated with natural rubber (NR) latex through a padding process, followed by passage through nip rollers to ensure uniform coating distribution. The coated samples were then dried in a hot-air oven at 60 °C for 30 min and conditioned at room temperature before further characterization.

### 2.8. Color Measurement and Color Fastness Evaluation

Color measurements of the dyed samples were performed using a spectrophotometer (GretagMacbeth LLC, Regensdorf, Switzerland) to determine color parameters based on the CIELAB color space, as well as the color strength (*K*/*S* value). Measurements were performed under illuminant D65, using a 10° standard observer, with both a specular and a UV filter applied. The average value from three measurements was recorded. In the CIELAB system, L* denotes lightness, ranging from 0 (black) to 100 (white); a* represents the red–green axis, where positive values indicate redness and negative values indicate greenness; b* represents the yellow–blue axis, where positive values indicate yellowness and negative values indicate blueness. The chroma (C*) describes color saturation or intensity. It is calculated from the a* and b* coordinates, while the hue angle (*h°*) represents the color shade and is defined by the angular position in the a*–b* color plane. Color fastness to light was evaluated according to ISO 105-B02:2014 [29], while color fastness to perspiration was measured in accordance with ISO 105-E04:1994 [30]. Color fastness to rubbing was evaluated according to ISO 105-X12:2016 [31].

### 2.9. FTIR Spectroscopy

Fourier transform infrared (FTIR) spectra of the plant-based leather samples dyed with lac dye were recorded using an FTIR spectrometer (Invenio R, Bruker, Karlsruhe, Germany) over the wavenumber range of 400–4000 cm^−1^, with a spectral resolution of 4 cm^−1^ and 64 scans. Measurements were performed in attenuated total reflectance (ATR) mode.

### 2.10. Moisture Management Test

The moisture management behavior of the samples was evaluated in accordance with AATCC Test Method 195-2011e2 using a Moisture Management Tester (MMT^®^, M290, SDL Atlas, Rock Hill, SC, USA). This test assesses the liquid moisture transport properties of textile materials by measuring parameters such as absorption rate, spreading speed, and accumulative one-way transport capability on both the top and bottom surfaces. The overall moisture management capacity (OMMC) and the corresponding moisture transport performance grades were automatically calculated by the MMT software based on the classification criteria defined in AATCC 195-2011e2. During testing, the NR–coated BSS side of the sample was placed in contact with the top sensor to evaluate the liquid barrier and waterproof performance of the coated surface.

### 2.11. Bursting Resistance Test

Bursting strength was measured in accordance with ISO 13938-1:2019 [32] using the hydraulic diaphragm method, and the maximum rupture pressure was recorded.

## 3. Results and Discussion

### 3.1. Chemical Composition of BSS Fibers

The chemical composition of the BSS fibers used in this study was determined to provide a fundamental understanding of the substrate. The results are summarized in Table 1. The BSS fibers exhibited a high holocellulose content (65.22%), comprising α-cellulose (45.09%) and hemicellulose (20.13%), along with a relatively low lignin content (5.63%) and extractives (26.82 wt%).

### 3.2. Morphology of BSS Fibers

**Figure 1:**
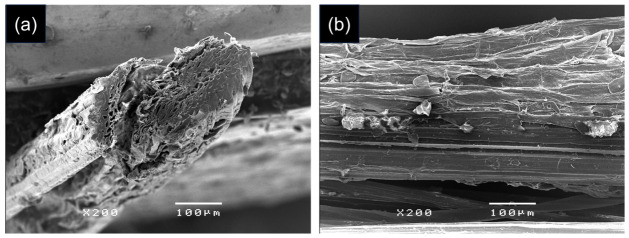
SEM images of BSS fibers: (**a**) cross-sectional view and (**b**) longitudinal view.

The morphology of BSS fibers was examined by SEM in both cross-sectional and longitudinal orientations (Figure 1). The cross-sectional image (Figure 1a) reveals an irregular and non-circular geometry with a rough and heterogeneous surface. A layered cell wall structure with visible fibrillar features and internal voids can be observed, which is characteristic of lignocellulosic materials. The apparent diameter of the BSS fibers was measured from 50 individual fibers and found to be 197 ± 27 µm. The longitudinal image (Figure 1b) shows an elongated and continuous structure with aligned fibrillar features along the fiber axis. The surface appears rough with longitudinal grooves and adhered particulates, reflecting the heterogeneous composition of the fiber. This anisotropic morphology, together with the presence of surface roughness and internal porosity, is expected to facilitate dye adsorption by providing accessible sites for interaction between the fiber, dye, and mordant system.

### 3.3. Lac Dyeing Properties of BSS

**Figure 2:**
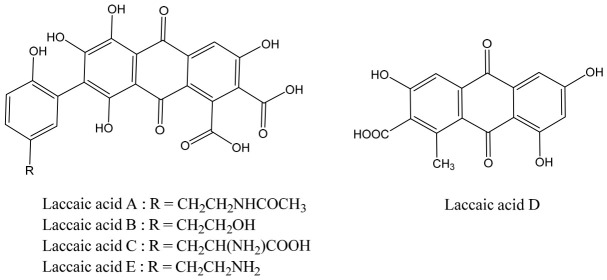
Chemical structures of laccaic acids (A–E), which are the principal colorant components of lac dye.

Lac dye consists of a mixture of at least five closely related laccaic acids derived from a 2-phenylanthraquinone backbone. These components include laccaic acids A, B, C, D, and E, among which laccaic acid A is the predominant constituent, accounting for 71–96%, followed by laccaic acid B (≈20%) [33]. The chemical structures of the laccaic acids are shown in Figure 2 [34].

The affinity of lac dye toward cellulose can be attributed to the presence of multiple hydroxyl (–OH), carbonyl (C=O), and carboxyl (–COOH) functional groups in laccaic acids. These functional groups facilitate extensive hydrogen bonding interactions with the abundant hydroxyl groups along the cellulose backbone.

#### 3.3.1. Effects of Dyeing Time

The color parameters of the lac-dyed BSS samples are presented in Table 2. It was observed that a dyeing time of 6 h produced the highest color strength (*K*/*S*) value among the samples. Extending the dyeing time beyond 6 h resulted in a decrease in *K*/*S* values, indicating reduced color strength. This reduction may be attributed to partial degradation of the lac dye and swelling of the BSS, which likely facilitated dye desorption from the substrate.

#### 3.3.2. Effects of Dye Concentration

The effects of dye concentration on the color properties are presented in Table 3. The results show that the *K*/*S* values increased sharply when the dye concentration was raised from 5% to 10% owf, whereas no further increase was observed at higher concentrations. This finding indicates that at a dye concentration of 10% owf, lac dye had already occupied most of the available binding sites on the BSS samples. At higher concentrations, additional dye molecules likely formed aggregates, which reduced their ability to penetrate and bind to the BSS substrate. Consequently, the *K*/*S* values reached a plateau despite further increases in dye concentration.

#### 3.3.3. Effects of Dye-Bath pH

The influence of dye-bath pH on the color strength of lac-dyed BSS samples is summarized in Table 4. The results indicate that acidic to mildly acidic conditions (pH 3–4.23) are favorable for lac dyeing of BSS, as relatively high *K*/*S* values were obtained within this pH range. The dyed samples also exhibited higher a* and C* values, reflecting a more intense reddish hue.

However, as the pH increased to neutral and alkaline conditions (pH 5–11), the *K*/*S* values decreased progressively. Previous studies have reported that the color of lac dye solutions appears orange, red, and violet under acidic, neutral, and alkaline conditions, respectively [35]. Lac dye contains laccaic acids as its primary colorants, which possess phenolic and carboxylic functional groups that readily undergo deprotonation. At pH values above 6, the ionized forms of laccaic acids become essentially colorless [35,36]. In addition, deprotonation generates negatively charged dye species, which further reduces dye uptake by the cellulose-rich BSS fibers. Since cellulose surfaces also carry negatively charged hydroxyl groups under neutral and alkaline conditions, electrostatic repulsion between the dye anions and the fiber surface reduces dye–fiber affinity, thereby explaining the observed decrease in color strength at higher pH values. Furthermore, lac dye has been reported to be chemically unstable at pH values above 9 due to instability of its quinonoid ring structure, which further contributes to color degradation under alkaline conditions [37].

Overall, the results confirm that lac dye exhibits optimal affinity toward BSS fibers under mildly acidic conditions, whereas dyeing at neutral or alkaline pH significantly reduces color strength due to decreased dye stability and reactivity. Although the highest *K*/*S* value was obtained at pH 3, such strongly acidic conditions may adversely affect the structural integrity of cellulose-rich fibers, potentially leading to fiber degradation or weakening. Therefore, a dye-bath pH of 4.23, corresponding to the natural pH of the lac dye solution without external adjustment, was selected as the optimal condition. This pH provided a balance between high color strength and preservation of fiber integrity and was employed for subsequent investigations.

#### 3.3.4. Effects of Mordant Concentration

Table 5 presents the color parameters and color strength of BSS dyed with natural lac dye using different mordant systems. These data were used to evaluate the influence of aluminum potassium sulfate (Al) and tannic acid (T), applied individually and in combination, on color depth and dye fixation efficiency.

**Figure 3:**
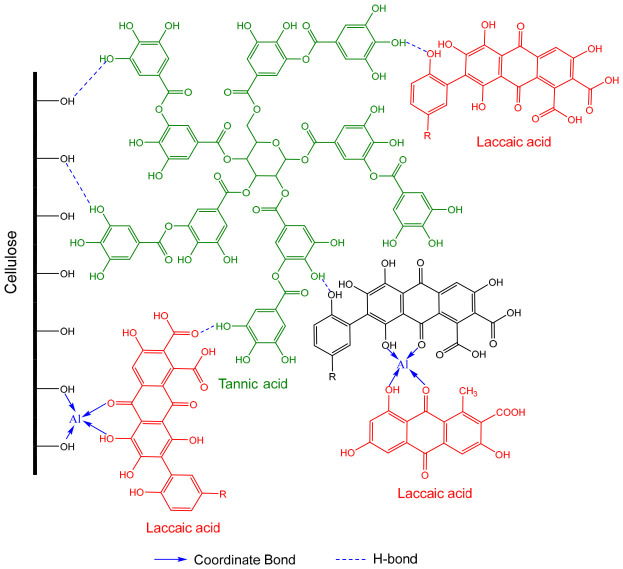
Schematic illustration of the proposed fixation mechanism of lac dye on cellulose in the presence of tannic acid and aluminum potassium sulfate, with tannic acid shown in green and laccaic acid shown in red.

The results indicate that the use of combined mordants produced higher color strength than single mordants alone. This enhancement can be attributed to the complementary roles of organic and metallic mordants in dye–fiber interactions. Tannic acid contains abundant hydroxyl (–OH) groups, which are capable of forming hydrogen bonds with both cellulose fibers and dye molecules. In addition, Al^3+^ ions can form coordination complexes with oxygen-donor functional groups of lac dye molecules, particularly carbonyl (C=O) and phenolic hydroxyl (–OH) groups thereby promoting the formation of a stable cellulose–mordant–dye complex. A schematic illustration of the proposed fixation mechanism of lac dye on cellulose using a mixed mordant system of tannic acid and aluminum potassium sulfate is shown in Figure 3.

Among all formulations, the sample pre-mordanted with tannic acid and aluminum potassium sulfate at equal concentrations of 5 g L^−1^ (T5Al5) exhibited the highest color strength, with a *K*/*S* value of 10.01. This synergistic interaction enhanced dye fixation and promoted greater color depth on the fiber surface.

**Figure 4:**
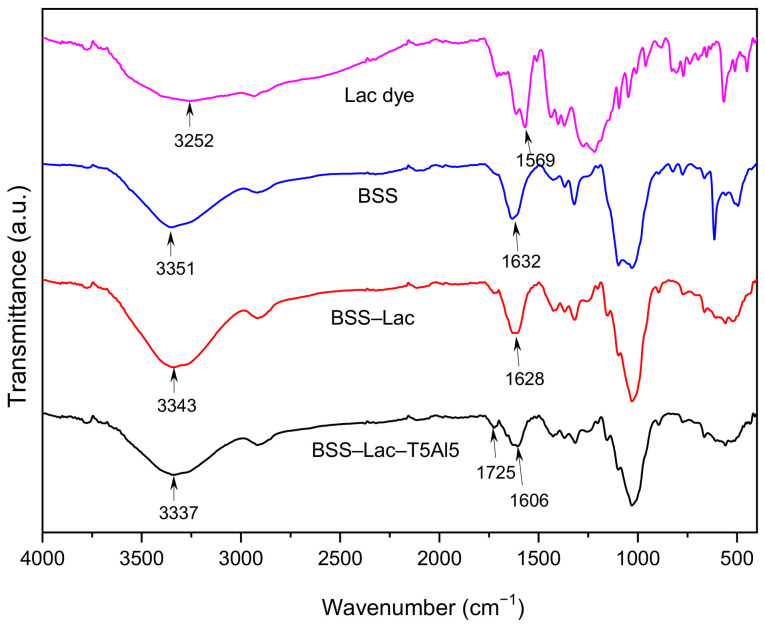
FTIR spectra of lac dye, untreated BSS, lac-dyed BSS (BSS–Lac), and mordanted lac-dyed BSS (BSS–Lac–T5Al5).

To further support the proposed dye–mordant–fiber interaction mechanism, FTIR and K/S spectral analyses were performed. The FTIR spectra (Figure 4) showed a slight shift in the band from approximately 1632 cm^−1^ in untreated BSS to 1628 cm^−1^ after lac dyeing (BSS–Lac). Such shifts in carbonyl (C=O) stretching bands are commonly associated with changes in the local chemical environment and may occur upon interaction or coordination with surrounding species [38]. In the mordanted sample (BSS–Lac–T5Al5), an additional band appeared at approximately 1606 cm^−1^, indicating a change in band position in the carbonyl-related region after mordanting. These changes are consistent with possible interactions involving Al^3+^ ions and oxygen-containing functional groups originating from the lac dye, tannic acid, and/or lignocellulosic components of BSS. In addition, tannic acid, which contains multiple phenolic hydroxyl groups, may contribute to the interaction network through hydrogen bonding with both the dye molecules and cellulose. However, due to overlapping contributions from the lignocellulosic substrate and polyphenolic components, precise band attribution remains limited.

**Figure 5:**
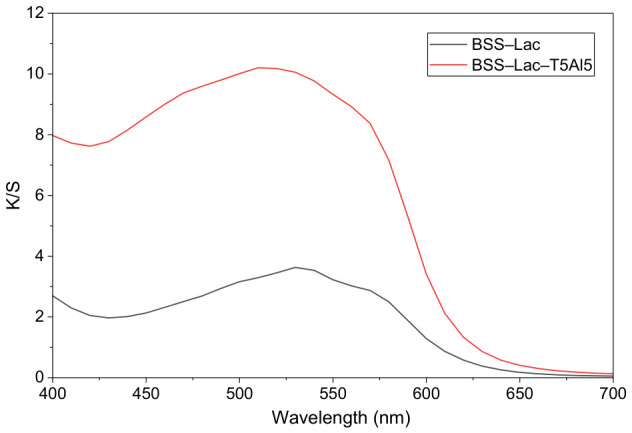
K/S spectra of lac-dyed BSS (BSS–Lac) and mordanted lac-dyed BSS (BSS–Lac–T5Al5) in the visible range (400–700 nm).

In addition, the *K*/*S* spectra (*K*/*S* vs. wavelength plot, Figure 5) revealed a shift in the maximum absorption wavelength (λ_max_) from 530 nm for lac-dyed BSS to 510 nm for mordanted lac-dyed BSS. This hypsochromic shift indicates a change in the electronic environment of the dye molecules, supporting the presence of dye–mordant–fiber interactions.

#### 3.3.5. Visual Appearance of BSS After Lac Dyeing

**Figure 6:**
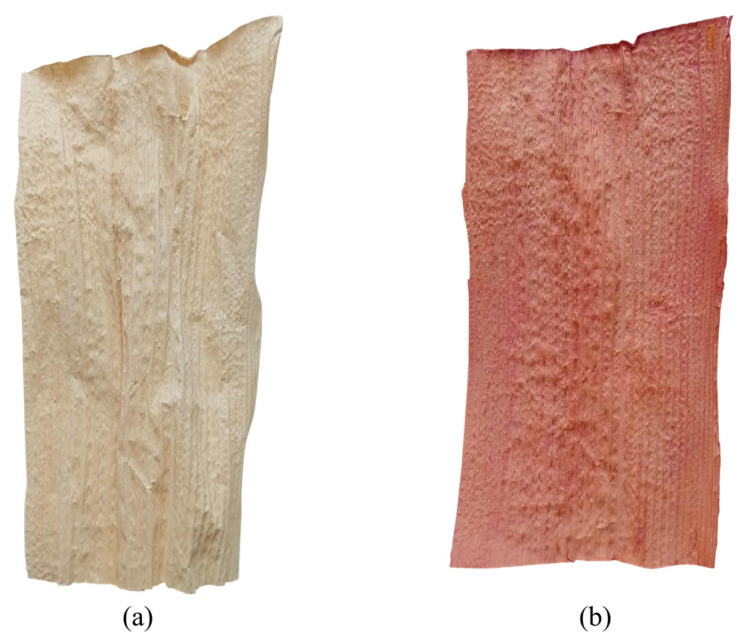
Photographic images of BSS samples: (**a**) BSS before dyeing; (**b**) BSS dyed with lac under optimum dyeing conditions.

The visual appearance of BSS samples before and after dyeing under the optimum lac dyeing conditions is presented in Figure 6. A clear color change from the natural pale beige of untreated BSS (Figure 6a) to a reddish tone after dyeing (Figure 6b) was observed, indicating successful coloration of the substrate.

The optimum dyeing conditions consisted of tannic acid and aluminum potassium sulfate at equal concentrations of 5 g L^−1^ (T5Al5), a dye concentration of 10% owf, pH 4.23, and a dyeing time of 6 h using the cold pad-batch technique. Under these conditions, the dyed sample exhibited relatively uniform color distribution over the surface, although slight variations in shade intensity were visible. This behavior may be attributed to the heterogeneous and porous structure of the BSS fibers, which can influence dye penetration and local dye uptake.

### 3.4. Indigo Dyeing Properties of BSS

**Figure 7:**
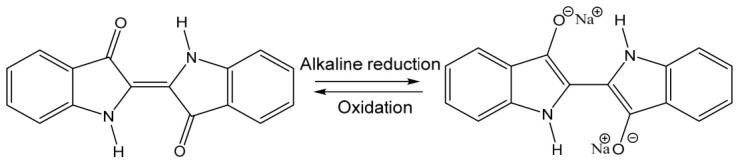
Redox mechanism of indigo dyeing showing the alkaline reduction of indigo to diionic leuco-indigo and its subsequent oxidation back to insoluble indigo.

The chemistry of indigo dyeing is governed by a redox mechanism, as illustrated in Figure 7 [24]. Under alkaline conditions (pH 11–14), water-insoluble indigo is reduced by reducing agents to form yellow, water-soluble leuco indigo [39]. The reduced dye exhibits substantivity toward cellulosic fibers, which allows diffusion into the fiber interior. Subsequent exposure to air during drying oxidizes leuco indigo back to its insoluble form, producing a stable blue coloration that becomes physically entrapped within the fiber matrix and is therefore resistant to washing [40]. The ionic form of leuco indigo is strongly dependent on pH. Under moderately alkaline conditions (pH 11–12), the monoionic species predominates and exhibits a higher affinity toward cellulose during vat dyeing. In contrast, under strongly alkaline conditions (pH > 13), the diionic form becomes dominant, which increases electrostatic repulsion between negatively charged dye species and the cellulose surface, thereby resulting in reduced dye uptake and lighter shades [24].

#### 3.4.1. Effects of Wet Indigo Content

As shown in Table 6, increasing the wet indigo concentration resulted in a corresponding increase in color strength. The highest *K*/*S* value of 13.43 was obtained at a wet indigo concentration of 500 g L^−1^, producing a deep blue shade. This behavior can be attributed to an optimal balance between the amount of indigo pigment and the reducing capacity of thiourea dioxide. Under this condition, a thiourea dioxide concentration of 80 g L^−1^ was sufficient to effectively reduce indigo to its soluble leuco-indigo form, thereby enhancing dye penetration and fixation within the BSS fibers. As a result, maximum dye uptake and color strength were achieved.

However, when the wet indigo concentration was further increased to 700 g L^−1^, a noticeable decrease in *K*/*S* values was observed. This reduction is attributed to an insufficient amount of reducing agent relative to the increased dye concentration. Under these conditions, indigo was not fully reduced in the dye bath, resulting in incomplete formation of leuco-indigo species. Consequently, dye diffusion into the BSS fibers was limited, leading to lower color strength despite the higher dye dosage.

#### 3.4.2. Effects of Sodium Hydroxide Concentration

Table 7 summarizes the effect of sodium hydroxide concentration on the indigo dyeing of BSS sheets, conducted under fixed conditions: 500 g L^−1^ wet indigo, 80 g L^−1^ thiourea dioxide, a liquor ratio of 1:50, and a reduction temperature of 30 °C for 30 min. Sodium hydroxide plays a crucial role in maintaining the alkaline environment required for the reduction of indigo and stabilization of leuco-indigo species [26]. In this study, a sodium hydroxide concentration of 30 g L^−1^ yielded the highest color strength, with a *K*/*S* value of 11.70. In contrast, lower concentrations of 10 and 20 g L^−1^ resulted in comparable but significantly reduced *K*/*S* values of 8.04 and 8.06, respectively, producing lighter blue shades. These sodium hydroxide concentrations corresponded to strongly alkaline conditions (pH ≈ 13.4–13.9), under which the diionic form of leuco-indigo species is expected to predominate. Therefore, the enhanced color strength observed at 30 g L^−1^ could not be explained solely by the ionic state of the dye. Instead, the higher alkali concentration likely promoted increased swelling of the cellulose-rich BSS sheets, thereby improving accessibility within the thick and compact substrate structure and enhancing dye diffusion and penetration. As a result, the positive effect of substrate swelling outweighed the reduced intrinsic affinity of the diionic dye species, leading to a higher overall color strength.

A slight weight loss of 2.94 ± 1.56% (*n* = 3) was observed after alkaline treatment at 30 g L^−1^ NaOH, suggesting partial removal of non-cellulosic components such as hemicellulose or extractives. This result indicates that although alkaline conditions may induce some degree of structural modification, no severe degradation of the cellulose backbone occurred under the applied conditions.

#### 3.4.3. Effects of Thiourea Dioxide Concentration

As shown in Table 8, the thiourea dioxide concentration significantly influenced the color strength and shade of indigo-dyed BSS samples. Increasing the reducing agent concentration from 10 to 40 g L^−1^ increased color strength, with the highest *K*/*S* value of 13.71 at 40 g L^−1^. This condition was accompanied by lower L* and more negative b* values, indicating a deeper bluish shade. These results suggest efficient reduction of indigo and effective diffusion of leuco-indigo into the fiber structure. At 60 g L^−1^, the color strength remained relatively high (*K*/*S* = 13.28). However, further increases in thiourea dioxide concentration led to a pronounced decline in *K*/*S* values, decreasing to 11.76 at 80 g L^−1^ and 7.71 at 100 g L^−1^. Such excessive reduction conditions likely destabilized leuco-indigo and reduced dye fixation during subsequent oxidation and rinsing steps.

Overall, a thiourea dioxide concentration of 40 g L^−1^ provided an optimal balance between effective indigo reduction and dye fixation, resulting in the highest color strength under the investigated conditions.

#### 3.4.4. Visual Appearance of BSS After Indigo Dyeing

**Figure 8:**
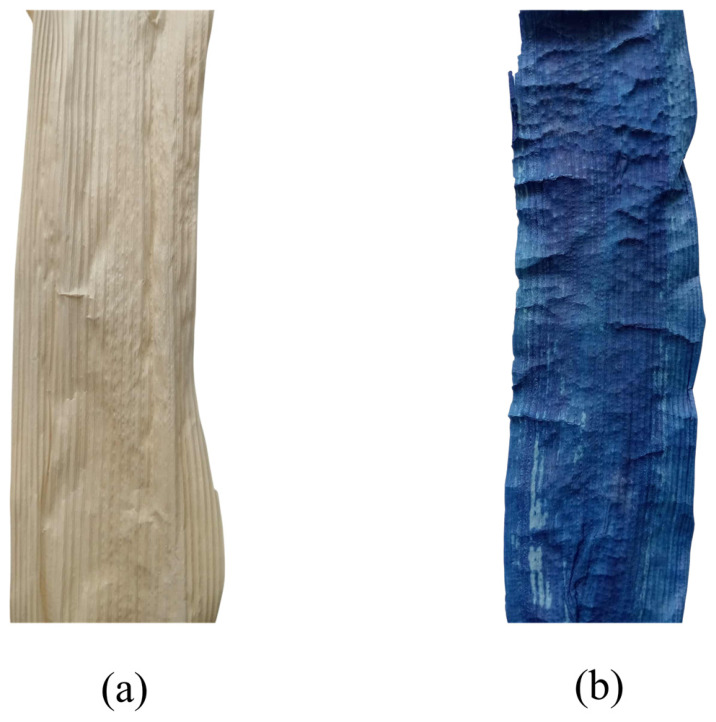
Photographic images of BSS samples: (**a**) BSS before dyeing; (**b**) BSS dyed with indigo under optimum dyeing conditions.

The visual appearance of BSS samples before and after dyeing under the optimum indigo dyeing conditions is presented in Figure 8. A distinct color change from the natural pale beige of untreated BSS (Figure 8a) to a deep blue color after dyeing (Figure 8b) was observed, indicating successful dye uptake.

The optimum dyeing conditions consisted of a thiourea dioxide concentration of 40 g L^−1^, NaOH at 30 g L^−1^, and a wet indigo concentration of 500 g L^−1^. Under these conditions, the indigo-dyed BSS exhibited a relatively uniform color distribution across the surface, although slight variations in shade intensity were observed. This may be attributed to the heterogeneous and porous structure of the BSS fibers, which influences dye penetration and the local deposition of indigo particles.

### 3.5. Color Fastness Results of BSS-Based Leather

The color fastness to perspiration of lac- and indigo-dyed BSS sheets coated with PP nonwoven fabric and NR latex was evaluated under both acidic and alkaline conditions. The results were assessed for color staining and color change, as summarized in Table 9. The color-staining ratings under both acidic and alkaline perspiration conditions were classified good to excellent, with ratings of at least 4–5, indicating that dye migration from the coated BSS sheets to adjacent fabrics was effectively suppressed.

In contrast, the color change ratings were relatively low, with a rating of 2, indicating a noticeable color change after perspiration exposure. This behavior was attributed to the intrinsic sensitivity of natural dyes to pH variations and moisture, even though the NR latex coating effectively limited dye leaching.

The color fastness to rubbing of the lac- and indigo-dyed BSS-based leather was evaluated under both dry and wet conditions. As shown in Table 10, both samples exhibited excellent rubbing fastness, achieving a rating of 5 in all cases, indicating minimal dye transfer during mechanical action.

The superior rubbing fastness observed for both dyes can be attributed to the presence of the NR latex coating, which likely acts as a protective barrier, physically encapsulating the dye molecules and enhancing their adhesion to the BSS substrate. This coating not only reduces surface dye migration but also improves resistance to frictional forces under both dry and moist conditions.

Notably, despite the relatively lower color change ratings observed after perspiration exposure, the rubbing fastness remained excellent. This suggests that the color change is more likely associated with intrinsic dye sensitivity to environmental conditions (e.g., pH and moisture), rather than dye detachment or surface loss. Therefore, the coating system effectively prevents dye transfer, even though some degree of shade alteration may still occur.

The color fastness to light of lac- and indigo-dyed BSS-based leather was evaluated based on color change, and the results are presented in Table 10. The findings indicate that the light fastness ratings were classified as fair, with ratings of 3–4 for the lac-dyed samples and 3 for the indigo-dyed samples. This moderate level of light fastness is characteristic of natural dyes, which are known to undergo photodegradation under prolonged exposure to light [41,42]. Although the natural rubber latex coating provided partial surface protection, it did not fully prevent photo-induced fading of the natural dye chromophores.

### 3.6. Bursting Resistance

The mechanical performance of lac- and indigo-dyed BSS-based leather was evaluated in terms of bursting strength, as summarized in Table 11. All specimens were prepared from BSS sheets laminated with polypropylene (PP) nonwoven fabric, with variations arising from dyeing treatment and the presence of a natural rubber (NR) latex coating. The bursting strength values of the lac-dyed and indigo-dyed coated samples were 51.8 and 54.8 psi, respectively, which were only slightly lower than that of the undyed and coated BSS sample at 56.6 psi. Notably, all coated samples exhibited substantially higher bursting strength than the control sample, defined as BSS sheets laminated with PP nonwoven fabric without dyeing or NR latex coating, which showed a bursting strength of 41.0 psi. These results indicate that NR latex coating effectively reinforced the structure. Overall, the findings demonstrate that the dyeing process did not significantly compromise the structural integrity of the BSS-based leather.

### 3.7. Moisture Management Properties

According to the results presented in Table 12, which summarizes the raw moisture management parameters obtained from the MMT test, all samples consisted of BSS sheets laminated with PP nonwoven fabric. The control sample consisted of undyed BSS sheets laminated with PP nonwoven fabric. In contrast, variations among the other samples were associated with the dyeing treatment and the presence of NR latex coating. All samples exhibited negative one-way transport index values and negligible overall moisture management capability (OMMC) values. These quantitative results indicate very slow liquid absorption, limited spreading, absence of effective one-way liquid transport, and no liquid penetration through the specimen thickness.

Based on these measured parameters, the moisture management performance was subsequently classified according to AATCC 195-2011e2, as reported in Table 13. Both the one-way transport index and the OMMC were rated Grade 1, indicating poor moisture management performance. This behavior suggests that the laminated BSS structure itself functioned as an effective moisture barrier.

As reported in AATCC 195-2011e2, materials exhibiting low OMMC values typically show limited moisture transmission and strong resistance to liquid penetration [43]. The inherent waterproof behavior of the laminated BSS structure is therefore advantageous for plant-based leather applications, where resistance to liquid penetration is essential to maintain appearance and durability.

## 4. Conclusions

In this study, the natural dyeing of banana pseudo-stem (BSS) sheets for plant-based leather applications was successfully demonstrated using low-temperature dyeing processes. The BSS sheets were dyed with either lac or indigo, then laminated onto a polypropylene (PP) nonwoven support, followed by coating with natural rubber (NR) latex to form a leather-like composite. For lac dyeing, a pre-mordanting step using tannic acid and aluminum potassium sulfate as a binary mordant system was employed. The optimal lac dyeing conditions were identified as a dyeing time of 6 h, a dye concentration of 10% owf, a pH of 4.23, tannic acid and aluminum potassium sulfate concentrations of 5 g L^−1^, and a liquor ratio (L/R) of 1:30.

Natural indigo dyeing was also performed, and the optimal conditions were determined to include a wet indigo concentration of 500 g L^−1^, a sodium hydroxide concentration of 30 g L^−1^, and a thiourea dioxide concentration of 40 g L^−1^, with dye reduction carried out at 30 °C for 30 min and a liquor ratio of 1:50.

The color fastness to perspiration of lac- and indigo-dyed samples exhibited good to excellent resistance to color staining under acidic and alkaline conditions. However, the color change ratings were low, reflecting the inherent sensitivity of natural dyes. The color fastness to rubbing was rated excellent under both dry and wet conditions. The color fastness to light was rated as fair. The bursting strength values of the dyed samples ranged from 51.8 to 54.8 psi, indicating adequate mechanical performance. In addition, the moisture management results showed negligible liquid penetration and poor moisture transport, confirming an inherent waterproof behavior of the laminated BSS-based composite structure. Overall, this study demonstrated a resource-efficient and environmentally conscious strategy for converting agricultural residues into value-added, sustainable plant-based leather alternatives.

## Figures and Tables

**Table 1:** Chemical constituents of BSS fibers.

Component	Content (wt%)
Total extractives	26.82
- Extractives (alcohol-benzene soluble)	10.68
- Alcohol solubility	1.84
- Hot water solubility	14.30
Holocellulose	65.22
- α-cellulose	45.09
- Hemicellulose	20.13
Lignin	5.63

Note: Minor deviations from a total of 100% may occur due to methodological differences and experimental uncertainty.

**Table 2:** Effect of dyeing time on the color properties of BSS sheets dyed with natural lac dye via the cold pad–batch process at a dye concentration of 10% owf, a liquor ratio of 1:30, and pH of 4.23.

Dyeing Time (h)	Color Parameter					*K*/*S*
	L*	a*	b*	C*	*h°*	
6	46.83	22.24	4.16	22.63	10.59	3.43 ± 0.13
12	54.39	25.02	4.17	25.39	9.49	2.29 ± 0.34
18	50.92	22.84	4.19	23.26	10.48	2.67 ± 0.18
24	52.84	34.64	11.40	36.47	18.16	3.08 ± 0.23

**Table 3:** Effect of dye concentration on the color properties of BSS sheets dyed with natural lac dye using the cold pad–batch technique at a dyeing time of 6 h, a liquor ratio of 1:30, and a pH of 4.23.

Dye Concentration (%owf)	Color Parameter					*K*/*S*
	L*	a*	b*	C*	*h°*	
5	60.32	21.70	−0.10	21.71	119.78	1.36 ± 0.27
10	45.89	23.89	1.18	23.96	122.45	3.71 ± 0.68
20	47.54	29.36	2.49	29.47	4.87	3.69 ± 0.14
30	47.85	29.79	1.45	29.86	2.73	3.64 ± 0.32

**Table 4:** Effect of dye-bath pH on the color properties of BSS sheets dyed with natural lac dye using the cold pad–batch technique at a dyeing time of 6 h, a liquor ratio of 1:30, and a dye concentration of 20% owf.

pH	Color Parameter					*K*/*S*
	L*	a*	b*	C*	*h°*	
3	52.11	27.49	1.57	27.54	3.22	2.63 ± 0.03
4.23	53.06	25.57	2.42	25.69	5.40	2.38 ± 0.10
5	56.33	24.30	1.47	24.35	3.44	1.88 ± 0.33
7	57.23	23.23	2.14	23.33	5.29	1.75 ± 0.29
9	56.69	23.73	1.06	23.77	122.52	1.80 ± 0.08
11	56.83	20.83	2.44	20.98	6.67	1.67 ± 0.19

**Table 5:** Effect of mordant type and concentration on the color properties of BSS sheets dyed with natural lac dye using the cold pad–batch technique at a dye concentration of 20% owf, a dyeing time of 6 h, a liquor ratio of 1:30, and a pH of 4.23.

Mordant	Color Parameter					*K*/*S*
	L*	a*	b*	C*	*h°*	
No mordant	47.54	29.36	2.49	29.47	4.87	3.69 ± 0.14
T5	42.87	28.98	5.58	28.57	11.25	5.23 ± 0.43
T10	42.52	30.25	5.07	30.68	9.51	5.33 ± 0.04
Al5	38.30	37.44	8.00	38.29	12.07	4.82 ± 0.30
Al10	45.68	39.16	11.60	40.85	16.47	5.58 ± 0.06
T5Al5	33.44	30.35	10.15	32.02	18.27	10.01 ± 0.18
T10Al5	39.63	35.66	6.12	35.57	9.94	7.68 ± 0.29
T5Al10	42.21	34.17	7.88	35.08	13.01	8.20 ± 0.08
T10Al10	37.15	24.61	2.93	24.79	6.80	6.78 ± 0.37

**Table 6:** Effect of wet indigo concentration on the color properties of BSS sheets dyed with natural indigo using thiourea dioxide at 80 g L^−1^, sodium hydroxide at 20 g L^−1^, a liquor ratio of 1:50, and reduction at 30 °C for 30 min.

Wet Indigo Content (g L^−1^)	Color Parameter					*K*/*S*
	L*	a*	b*	C*	*h°*	
100	38.67	−4.70	−4.02	11.99	246.90	6.41 ± 0.45
200	22.98	1.73	−3.12	3.60	298.67	12.82 ± 1.27
300	23.72	1.34	−3.88	4.20	291.59	12.26 ± 1.63
400	27.38	1.05	−4.57	4.88	283.82	10.96 ± 0.50
500	25.06	1.25	−1.74	3.47	299.14	13.43 ± 0.41
700	29.16	0.37	−3.46	3.32	277.00	8.63 ± 0.59
900	28.80	0.57	−4.29	5.41	282.23	8.70 ± 0.89

**Table 7:** Effect of sodium hydroxide concentration on the natural indigo dyeing of BSS sheets using thiourea dioxide at 80 g L^−1^, a wet indigo concentration of 500 g L^−1^, a liquor ratio of 1:50, and reduction at 30 °C for 30 min.

Sodium Hydroxide (g L^−1^)	Color Parameter					*K*/*S*
	L*	a*	b*	C*	*h°*	
10	30.25	1.04	−7.80	7.90	277.89	8.36 ± 0.34
20	30.54	−0.06	−5.32	6.00	269.22	8.06 ± 0.34
30	26.71	0.15	−3.56	8.02	271.01	11.17 ± 0.78

**Table 8:** Effect of thiourea dioxide concentration on the natural indigo dyeing of BSS sheets using a wet indigo concentration of 500 g L^−1^, sodium hydroxide at 20 g L^−1^, a liquor ratio of 1:50, and reduction at 30 °C for 30 min.

Thiourea Dioxide Concentration (g L^−1^)	Color Parameter					*K*/*S*
	L*	a*	b*	C*	*h°*	
10	23.39	2.12	−1.65	3.02	317.68	13.08 ± 0.57
40	22.49	1.04	−3.62	4.09	285.79	13.71 ± 0.81
60	26.95	0.17	−3.05	3.85	274.12	13.28 ± 0.31
80	24.06	1.05	−3.61	3.77	286.84	11.76 ± 0.71
100	29.71	0.85	−4.90	4.39	281.22	7.71 ± 0.66

**Table 9:** Color fastness to perspiration of BSS-based leather evaluated according to ISO 105-E04:1994.

Condition	Sample	Color Change Rating	Color Staining Rating					
			Acetate	Cotton	Nylon	Polyester	Acrylic	Wool
Acid	Lac-dyed	2	4–5	4	4–5	4–5	4–5	4
	Indigo-dyed	2	4–5	4–5	5	5	5	5
Alkaline	Lac-dyed	2	4–5	4	4–5	4–5	4	4–5
	Indigo-dyed	2	4–5	4–5	4–5	5	5	5

**Table 10:** Color fastness properties of BSS-based leather evaluated according to ISO 105-X12:2016 (rubbing fastness) and ISO 105-B02:2014 (light fastness).

Sample	Rubbing (Dry)	Rubbing (Wet)	Light Fastness
Lac-dyed	5	5	3–4
Indigo-dyed	5	5	3

**Table 11:** Bursting strength of BSS-based leather evaluated according to ISO 13938-1:2019.

Sample	Bursting Strength (psi)
Control (Undyed/Uncoated)	41.0 ± 3.94
Undyed/Coated	56.6 ± 3.05
Lac-dyed/Coated	51.8 ± 5.12
Indigo-dyed/Coated	54.8 ± 3.42

**Table 12:** Moisture management properties of BSS-based leather evaluated using MMT according to AATCC 195-2011e2.

Sample	Top Absorption Rate (%/s)	Bottom Absorption Rate (%/s)	Top Spreading Speed (mm/s)	Bottom Spreading Speed (mm/s)	One-Way Transport Index (%)	OMMC
Control (Undyed/Uncoated)	79.26 ± 89.69	0.97 ± 1.68	0.52 ± 0.23	0.00 ± 0.00	−1086.83 ± 213.74	0.00 ± 0.00
Undyed/Coated	176.85 ± 28.15	3.20 ± 0.30	0.59 ± 0.03	0.00 ± 0.00	−1490.75 ± 414.38	0.00 ± 0.00
Lac-dyed/Coated	199.01 ± 51.93	0.92 ± 1.59	0.52 ± 0.06	0.00 ± 0.00	−1437.85 ± 434.17	0.00 ± 0.00
Indigo-dyed/Coated	74.83 ± 60.40	0.00 ± 0.00	0.59 ± 0.06	0.00 ± 0.00	−1402.38 ± 190.86	0.00 ± 0.00

**Table 13:** Moisture management grading of BSS-based leather evaluated according to AATCC 195-2011e2, with grades assigned based on the classification criteria defined in AATCC 195, where 1 represents poor performance, and 5 represents excellent performance.

Sample	Top Absorption Grade	Bottom Absorption Grade	Spreading Grade	One-Way Transport Grade	OMMC Grade
Control (Undyed/Uncoated)	3	1	1	1	1
Undyed/Coated	5	1	1	1	1
Lac-dyed/Coated	5	1	1	1	1
Indigo-dyed/Coated	3	1	1	1	1

## Data Availability

The raw data supporting the conclusions of this article will be made available by the authors on request.
